# Desfechos Clínicos e Mortalidade em Pacientes com Cardioversor-Desfibrilador Implantável para Prevenção Primária

**DOI:** 10.36660/abc.20240348

**Published:** 2025-02-11

**Authors:** Ahmet Anıl Başkurt, Sema Güneri, Reşit Yiğit Yılancıoğlu, Oğuzhan Ekrem Turan, Emin Evren Özcan

**Affiliations:** 1 Department of Cardiology Bakırçay University Faculty of Medicine İzmir Turquia Department of Cardiology, Bakırçay University Faculty of Medicine, İzmir – Turquia; 2 Department of Cardiology Dokuz Eylul University Faculty of Medicine İzmir Turquia Department of Cardiology, Dokuz Eylul University Faculty of Medicine, İzmir – Turquia

**Keywords:** Desfibriladores Implantáveis, Insuficiência Cardíaca, Prevenção Primária

## Abstract

**Fundamento:**

O cardioversor-desfibrilador implantável (CDI) é indicado para prevenção primária em pacientes com fração de ejeção do ventrículo esquerdo (FEVE) ≤ 35% e insuficiência cardíaca classe II ou III da New York Heart Association, apesar de 3 meses de terapia médica otimizada. No entanto, os estudos que apoiam essa recomendação têm mais de 20 anos e podem não refletir as características dos pacientes modernos com insuficiência cardíaca.

**Objetivos:**

Avaliar retrospectivamente os pacientes que receberam CDI para prevenção primária.

**Métodos:**

As taxas de morte por todas as causas e morte súbita foram comparadas em pacientes que receberam CDI entre 1º de janeiro de 2015 e 1º de março de 2020 e aqueles que não aceitaram o CDI. As variáveis foram analisadas em um intervalo de confiança de 95%, e p < 0,05 foi considerado significativo.

**Resultados:**

Ao comparar as taxas de mortalidade entre pacientes com e sem CDI, 67 de 228 pacientes (29,4%) no grupo CDI e 39 de 150 pacientes (26%) no grupo controle apresentaram mortalidade por todas as causas (p = 0,473). Idade, FEVE, valor de BNP e hospitalização foram considerados preditores independentes de mortalidade por todas as causas. Pacientes com BNP acima de 508,5, FEVE abaixo de 24,5% e idade acima de 68,5 anos tiveram uma mortalidade por todas as causas 25 vezes maior. A doença arterial coronária não foi considerada um fator de risco independente. A sobrevida no grupo controle foi estatisticamente significativamente melhor nos primeiros meses. Embora não tenha havido diferença estatística em longo prazo, a sobrevida foi numericamente melhor grupo CDI. Isso pode ser atribuído ao fato de que os implantes de CDI foram realizados em pacientes com piores condições clínicas. A taxa maior de sobrevida observada em pacientes com CDI pode ser devida ao fato de que eles vieram para o controle do dispositivo e permaneceram em acompanhamento.

**Conclusões:**

Com os avanços no tratamento da insuficiência cardíaca, o implante de CDI deve ser realizado em pacientes selecionados.

## Graphical abstract

**Figura Central:**
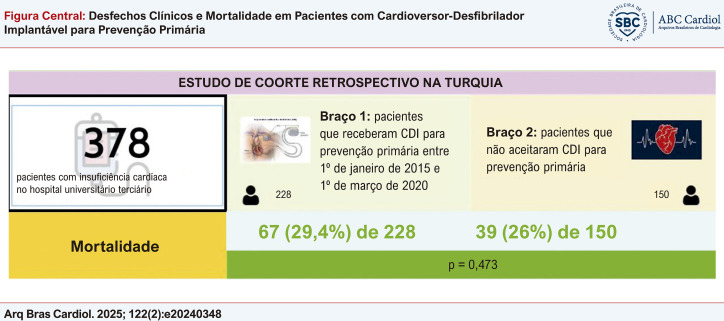
Desfechos Clínicos e Mortalidade em Pacientes com Cardioversor-Desfibrilador Implantável para Prevenção Primária

**Figura Central:** Desfechos Clínicos e Mortalidade em Pacientes com Cardioversor-Desfibrilador Implantável para Prevenção Primária

*CDI: cardioversor-desfibrilador implantável.*

## Introdução

Em pacientes com fração de ejeção do ventrículo esquerdo (FEVE) abaixo de 35% e insuficiência cardíaca classe II ou III da New York Heart Association (NYHA), com pelo menos 3 meses de terapia médica otimizada, deve ser considerada a prevenção primária com um cardioversor-desfibrilador implantável (CDI).^1^ No entanto, os estudos nos quais essa recomendação se baseia têm mais de 20 anos, podendo não refletir as características e o tratamento de pacientes com insuficiência cardíaca hoje. Portanto, os efeitos do CDI na prevenção primária podem ter mudado.

As taxas de choque e de mortalidade têm diminuído em pacientes com implante de CDI devido ao desenvolvimento do sistema de saúde, acesso mais fácil a médicos e disponibilidade de novos tratamentos para insuficiência cardíaca. Com as novas opções de tratamento disponíveis para insuficiência cardíaca, a mortalidade geral nesse grupo de pacientes diminuiu gradualmente. Além disso, o implante de CDI para prevenção primária tem sido questionado após ensaios de insuficiência cardíaca usando inibidores de SGLT2 e inibidores da neprilisina e do receptor de angiotensina (INRA).^2-4^ Hoje, muitos pacientes com CDI não recebem nenhum choque. O objetivo do nosso estudo foi avaliar retrospectivamente pacientes que receberam CDI para prevenção primária.

## Materiais e métodos

Utilizamos arquivos de hospitais universitários terciários, registros de pacientes e informações de histórico clínico do sistema Probel. Nesse contexto, realizamos a triagem de 504 pacientes submetidos a implante de CDI de janeiro de 2015 a março de 2020 e para os quais obtivemos dados completos. Determinamos que 289 desses pacientes receberam CDI para prevenção primária. Testes genéticos e diagnósticos revelaram que 12 desses 289 pacientes tinham canalopatia e 49 pacientes foram submetidos à substituição do gerador. Portanto, 228 pacientes tinham insuficiência cardíaca com baixa FEVE e foram submetidos a implante de CDI para prevenção primária. Durante o mesmo período, o estudo incluiu 150 pacientes como grupo controle, que tinham indicação para implante de CDI para prevenção primária, mas não aceitaram o tratamento. O Comitê de Ética em Pesquisa Não Invasiva da Universidade Dokuz Eylül aprovou o protocolo do estudo (número de aprovação: 2020/18-02, data: 10 de agosto de 2020).

### Análise estatística

Os dados foram avaliados usando o SPSS 22.0 (IBM Corporation, Armonk, NY, EUA). A distribuição normal das variáveis foi avaliada com o teste de Kolmogorov–Smirnov, e a homogeneidade da variância foi avaliada com o teste de Levene. Os dados determinados pela medição foram apresentados como média e desvio padrão para aqueles com distribuição normal e como mediana e intervalo interquartil para aqueles que não foram distribuídos normalmente. O teste t não pareado ou teste U de Mann–Whitney (para valor de BNP, hemoglobulina, creatinina) foi usado na análise estatística desses dados de acordo com a normalidade dos dados. Variáveis categóricas foram apresentadas como frequências absolutas e relativas, sendo usado o teste qui-quadrado ou teste exato de Fisher (para morte súbita), conforme apropriado. As variáveis foram analisadas em um intervalo de confiança de 95% e p < 0,05 foi considerado significativo. A análise da característica de operação do receptor (ROC) foi usada para cálculos de área sob a curva. A regressão logística multinomial foi usada para determinar preditores independentes.

## Resultados

Dos 228 pacientes com CDI para prevenção primária, 175 (76,8%) eram do sexo masculino. A idade média dos pacientes foi de 65,63 (11,94) anos. O período médio de acompanhamento foi de 39,45 (18,89) meses. A duração média da hospitalização para o procedimento foi de 5,49 (3,99) dias. A Tabela 1 resume as características demográficas dos pacientes.

**Tabela 1:** – Características demográficas dos pacientes

	Grupo CDI (n = 228)	Grupo controle (n:150)	Valor p
**Idade, anos, média ± DP**	65,63 (11,94)	66,55 (12,78)	0,476
**Sexo, masculino, n(%)**	175 (76,8%)	107 (71,3%)	0,236
**Doença arterial coronária, n(%)**	135 (59,2%)	97 (64,7%)	0,286
**Hipertensão, n(%)**	145 (63,6%)	106 (70,7%)	0,155
**Diabetes mellitus, n(%)**	79 (34,6%)	45 (30,0%)	0,346
**Doença renal crônica, n(%)**	52 (22,8%)	26 (17,3%)	0,198
**DPOC, n(%)**	22 (9,6%)	9 (6,0%)	0,206
**Fibrilação atrial, n(%)**	60 (26,3%)	25 (16,7%)	**0,028**
**Acompanhamento, meses, média ± DP**	39,45 (18,89)	38,89 (11,61)	0,724
**Beta bloqueador, n(%)**	222 (97,4%)	144 (96,0%)	0,552
**IECA, BRA, INRA, n(%)**	189 (82,9%)	130 (86,7%)	0,323
**ARM, n(%)**	182 (79,8%)	127 (84,7%)	0,233

*ARM: antagonista do receptor mineralocorticoide; BRA: bloqueador do receptor da angiotensina; CDI: cardioversor-desfibrilador implantável; DP: desvio padrão; DPOC: doença pulmonar obstrutiva crônica; IECA: inibidor da enzima conversora de angiotensina; INRA: inibidor da neprilisina e do receptor de angiotensina-neprilisina.*

A análise dos achados ecocardiográficos transtorácicos de pacientes com CDI revelou uma FEVE média de 24,30% (6,19%). Por outro lado, no grupo controle, a FEVE média foi de 30,77% (4,87%). As Tabelas 2 e 3 exibem os achados ecocardiográficos e os dados laboratoriais dos pacientes.

**Tabela 2:** – Dados ecocardiográficos dos pacientes

	Grupo CDI (n = 228)	Grupo controle (n = 150)	Valor p
**FEVE, % ± SD**	24,30% (6,19)	30,77% (4,87)	**< 0,0001**
**DDVE, cm ± SD**	6,00 (0,81)	5,54 (0,70)	**< 0,0001**
**DSVE, cm ± SD**	5,00 (0,91)	4,17 (0,92)	**< 0,0001**
**AE, cm ± SD**	4,53 (0,62)	4,31 (0,69)	**< 0,001**
**PSAP, mmHg ± SD**	31,69 (20,09)	32,93 (15,72)	0,505

*AE: átrio esquerdo; CDI: cardioversor-desfibrilador implantável; DDVE: diâmetro diastólico final do ventrículo esquerdo; DP: desvio padrão; DSVE: diâmetro sistólico final do ventrículo esquerdo; FEVE: fração de ejeção do ventrículo esquerdo; PSAP: pressão sistólica da artéria pulmonar.*

**Tabela 3:** – Dados laboratoriais dos pacientes

	Grupo CDI (n = 228)	Grupo controle (n = 150)	Valor p
**Hemoglobina gr/dL**	12,77 (1,80)	12,91 (1,93)	0,467
**Creatinina mg/dL**	1,04 (0,62)	0,89 (0,71)	**0,001**
**Na (mmol/L)**	137,74 (2,87)	138,53 (3,61)	**0,025**
**K (mmol/L)**	4,36 (0,50)	4,15 (0,46)	**< 0,0001**
**BNP pg/m**	421,00 (114,68)	415,50 (719,17)	0,932

*BNP: peptídeo natriurético tipo B; CDI: cardioversor-desfibrilador implantável; K: potássio; Na: sódio.*

Ao comparar as taxas de mortalidade entre pacientes com e sem CDI, 67 de 228 pacientes (29,4%) no grupo CDI e 39 de 150 pacientes (26%) no grupo controle apresentaram mortalidade por todas as causas (p = 0,473). Verificamos que 2 pacientes no grupo CDI e 8 pacientes no grupo controle tiveram morte súbita (p = 0,017; Tabelas 4 e 5).

**Tabela 4:** – Mortalidade por todas as causas nos grupos CDI e controle

		Grupo controle n = 150	Grupo CDI n = 228
**Mortalidade**	Sobrevida	111 (74%)	161 (70%)
	Óbito	39 (26%)	67 (30%)
**Total**		150	228

*CDI: cardioversor-desfibrilador implantável.*

**Tabela 5:** – Morte súbita nos grupos CDI e controle

		Grupo controle n = 150	Grupo CDI n = 228
**Morte súbita**	Sim	8 (5,3%)	2 (0,9%)
	Não	142 (94,7%)	226 (99,1%)
**Total**		150	228

*CDI: cardioversor-desfibrilador implantável.*

Analisamos os preditores de mortalidade por todas as causas em pacientes com CDI. Não houve diferença estatisticamente significativa entre pacientes com e sem mortalidade em termos de sexo ou doença arterial coronária. Idade, diabetes mellitus e insuficiência renal crônica foram estatisticamente significativamente maiores em pacientes com mortalidade (Tabela 6).

**Tabela 6:** – Dados demográficos de pacientes com CDI de acordo com sobrevida ou morte

	Morte n = 67	Sobrevida n = 161	Valor p
**Idade, anos, média ± DP**	70,68 (10,51)	63,57 (11,9)	**< 0,0001**
**Sexo, masculino, n(%)**	50 (74,6%)	125 (74,6%)	0,624
**Doença arterial coronária, n(%)**	45 (67,2%)	90 (55,9%)	0,115
**Hipertensão, n(%)**	45 (67,2%)	100 (62,1%)	0,470
**Diabetes mellitus, n(%)**	30 (44,8%)	49 (30,4%)	**0,038**
**Doença renal crônica, n(%)**	24 (35,8%)	28 (17,4%)	**0,003**
**DPOC, n(%)**	7 (10,4%)	15 (9,3%)	0,792
**Fibrilação atrial, n(%)**	27 (40,3%)	33 (20,5%)	**0,002**
**Hospitalização por IC descompensada, n(%)**	28 (41,8%)	23 (14,3%)	**< 0,0001**
**Classe NYHA > 2, n(%)**	26 (38,8%)	28 (17,4%)	**0,001**

*CDI: cardioversor-desfibrilador implantável; DPOC: doença pulmonar obstrutiva crônica; IC: insuficiência cardíaca; NYHA: New York Heart Association.*

Ao examinar os parâmetros ecocardiográficos transtorácicos e os resultados laboratoriais, verificamos que os pacientes com mortalidade apresentaram menor FEVE, maior tamanho do átrio esquerdo e pressão arterial pulmonar sistólica mais alta (Tabela 7). Houve uma diferença estatisticamente significativa entre o valor mediano do BNP entre os grupos (Tabela 8).

**Tabela 7:** – Dados ecocardiográficos de pacientes com CDI de acordo com sobrevida ou morte

	Morte n = 67	Sobrevida n = 161	Valor p
**FEVE, % ± DP**	21,75 (5,47)	25,36 (6,2)	**< 0,0001**
**DDVE, cm ± DP**	6,1 (0,70)	5,9 (0,84)	0,077
**DSVE, cm ± DP**	5,24 (0,86)	4,91 (0,91)	**0,013**
**AE, cm ± DP**	4,80 (0,58)	4,4 (0,60)	**< 0,0001**
**PSAP, mmHg ± DP**	36,75 (23,73)	29,59 (18,02)	**0,029**

*AE: átrio esquerdo; CDI: cardioversor-desfibrilador implantável; DDVE: diâmetro diastólico final do ventrículo esquerdo; DP: desvio padrão; DSVE: diâmetro sistólico final do ventrículo esquerdo; FEVE: fração de ejeção do ventrículo esquerdo; PSAP: pressão sistólica da artéria pulmonar.*

**Tabela 8:** – Dados laboratoriais de pacientes com CDI de acordo com sobrevida ou morte

	Morte n = 67	Sobrevida n = 161	Valor p
**Hemoglobina gr/dL**	12,29 (2,0)	13,0 (1,70)	**0,009**
**Creatinina mg/dL**	1,36 (0,61)	1,12 (0,61)	**< 0,0001**
**Na (mmol/L)**	137,00 (2,80)	138,06 (2,85)	**0,008**
**K (mmol/L)**	4,38 (0,57)	4,36 (0,48)	0,877
**BNP pg/m (n = 177)**	1424,44 (1384,83)	537,82 (783,80)	**< 0,0001**

*BNP: peptídeo natriurético tipo B; CDI: cardioversor-desfibrilador implantável; K: potássio; Na: sódio.*

### Preditores de mortalidade na análise de regressão logística multinomial

A análise de regressão logística multinomial incluiu variáveis que podem influenciar a mortalidade no modelo usando o método “*enter*”. Essas variáveis incluíram idade, doença arterial coronária, diabetes mellitus, insuficiência renal crônica, ritmo basal, hospitalização por insuficiência cardíaca, classe NYHA > 2, complicações e valor do BNP. A análise revelou que idade, FEVE, valor do BNP e hospitalização por descompensação foram fatores independentes para mortalidade por todas as causas. A mortalidade foi 3,4 vezes maior em pacientes hospitalizados com insuficiência cardíaca descompensada antes do procedimento. A doença arterial coronária não foi um fator de risco independente (Tabela 9).

**Tabela 9:** – Resultados da análise de regressão logística multinomial

	B	SE	Wald	OR (IC 95%)	p
**Idade**	0,067	0,021	9,799	1,069 (1,025 a 1,114)	**0,002**
**FEVE**	–0,097	0,036	7,411	0,907 (0,846 a 0,973)	**0,006**
**DAC**	0,032	0,409	0,006	1,033 (0,463 a 2,301)	0,937
**DM**	0,530	0,397	1,782	1,699 (0,780 a 3,698)	0,182
**DRC**	–0,181	0,484	0,140	0,834 (0,323 a 2,155)	0,709
**FA**	0,306	0,414	0,547	1,358 (0,603 a 3,060)	0,460
**Hospitalização por insuficiência cardíaca descompensada**	1,211	0,433	7,820	3,355 (1,436 a 7,839)	**0,005**
**Classe NYHA > 2**	0,007	0,447	0,000	1,007 (0,420 a 2,418)	0,987
**BNP**	0,001	0,000	6,175	1,001 (1,000 a 1,001)	**0,013**

*BNP: peptídeo natriurético tipo B; DAC: doença arterial coronária; DM: diabetes mellitus; DRC: doença renal crônica; FEVE: fração de ejeção do ventrículo esquerdo; IC: intervalo de confiança; NYHA: New York Heart Association; OR: razão de chances; SE: erro padrão.*

Realizamos análise ROC para essas variáveis, encontrando valores preditivos de 68,5 anos com sensibilidade de 62% e especificidade de 62% para idade; 24,5% com sensibilidade de 54% e especificidade de 63% para FEVE; e 508,5 com sensibilidade de 69% e especificidade de 69% para valor de BNP.

A mortalidade por todas as causas dos pacientes com valor de BNP acima de 508,5, valor de FEVE abaixo de 24,5% e idade maior que 68,5 anos foi 25 vezes maior do que nos outros pacientes (Tabela 10).

**Tabela 10:** – Resultados da análise de regressão logística univariada

	B	SE	Wald	OR (IC 95%)	p
**Idade < 68,5 anos BNP < 508,5 FEVE > 24,5%**	–3,243	1,029	9,938	0,039 (0,005 a 0,293)	0,002

*BNP: peptídeo natriurético tipo B; FEVE: fração de ejeção do ventrículo esquerdo; IC: intervalo de confiança; OR: razão de chances; SE: erro padrão.*

**Figura 1:**
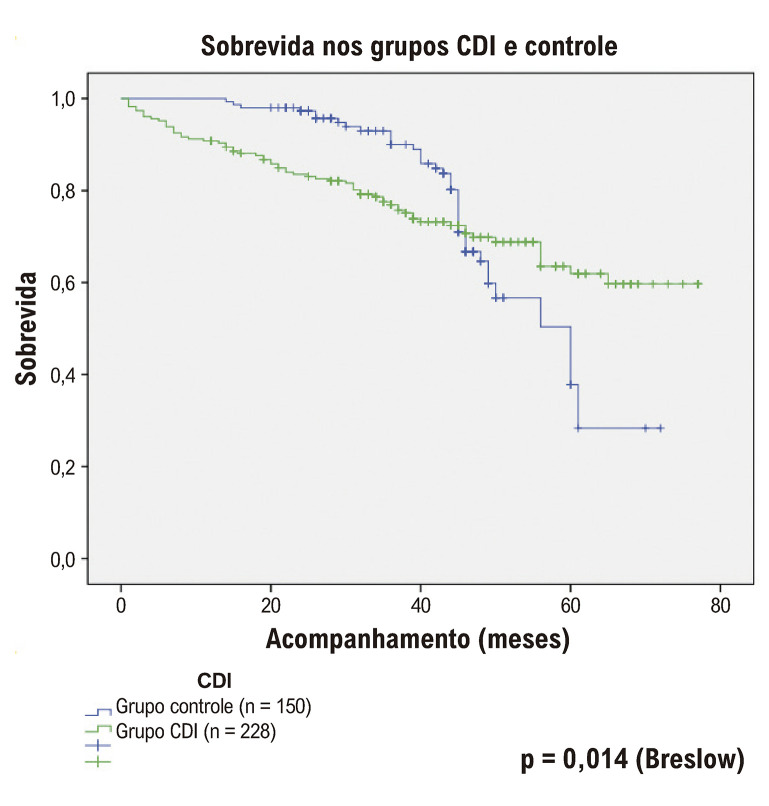
– Sobrevida nos grupos CDI e controle. CDI: cardioversor-desfibrilador implantável.

Quando as curvas de sobrevida dos dois grupos foram avaliadas, a sobrevida no grupo controle foi estatisticamente significativamente melhor nos primeiros meses em comparação ao grupo com CDI. No mês 44, as curvas de sobrevida dos dois grupos foram cruzadas. Embora não tenha havido diferença estatística em longo prazo, a sobrevida foi numericamente melhor no grupo CDI. A sobrevida média foi de 59 meses no grupo com CDI e 55 meses no grupo controle (Figura 1).

Isso pode ser atribuído ao fato de que os implantes de CDI foram realizados em pacientes com piores condições clínicas. A longo prazo, acreditamos que os pacientes com CDI implantado tiveram uma taxa de sobrevida maior porque eles vieram para o controle do dispositivo e permaneceram em acompanhamento.

## Discussão

Embora não tenha havido diferença significativa entre os pacientes com CDI e o grupo controle em termos de mortalidade por todas as causas, uma diferença estatisticamente significativa foi observada em termos de morte súbita (p = 0,017), que foi observada em 2 pacientes no grupo CDI e em 8 pacientes no grupo controle. Essa diferença estatística pode ser devida ao baixo número de eventos.

Uma metanálise publicada examinou 12 ensaios randomizados de insuficiência cardíaca com baixa FEVE ao longo de um período de 20 anos de 1995 a 2014 para risco de morte cardíaca súbita. Um total de 40.195 pacientes foram incluídos nos estudos. A incidência anual de morte cardíaca súbita foi de 6,5% no RALES,^5^ primeiro estudo que abrangeu esse período, e 3,3% no estudo PARADIGM-HF mais recente.^6^ Houve uma redução de 44% na taxa de morte cardíaca súbita em um período de 20 anos (*hazard ratio*: 0,56; intervalo de confiança de 95%: 0,33 a 0,93; p = 0,03). A incidência cumulativa de morte cardíaca súbita em 90 dias foi de 2,4% em estudos mais antigos e 1,0% em estudos recentes.^7^

Quando analisamos os estudos realizados de maneira semelhante ao nosso estudo, verificamos que 45.000 pacientes com CDI para prevenção primária foram estudados nos EUA. O estudo identificou os seguintes 7 preditores de mortalidade, que foram abreviados com as letras “SHOCKED”: idade ≥ 75 anos, insuficiência cardíaca, fibrilação atrial, doença pulmonar obstrutiva crônica, doença renal crônica, FEVE ≤ 20% e diabetes. No grupo de teste do modelo, usando o modelo SHOCKED, a mortalidade em 3 anos foi de 65% em pacientes no grupo com o risco 10% mais alto.^8^ Outro estudo publicado em 2012 incluiu 2.717 pacientes. Nessa publicação, doença arterial periférica, idade ≥ 70 anos, creatinina >2 mg/dL e FEVE ≤ 20% foram determinados como critérios de risco. Em pacientes com ≥ 3 desses critérios de risco, a mortalidade foi considerada 4 vezes maior (16,5% versus 3,4%) em comparação com pacientes com < 3 critérios.^9^ Outro estudo semelhante foi publicado em 2012. Incluindo 900 pacientes, foi desenvolvido em escore denominado FADES no qual NYHA > III, idade avançada, diabetes mellitus, FEVE ≤ 25% e tabagismo foram encontrados como critérios de risco.^10^ Os resultados do nosso estudo são semelhantes aos dessas publicações.

Outro estudo examinou o impacto da carga de insuficiência cardíaca e da carga de condições comórbidas na sobrevida de pacientes com CDI para prevenção primária atendidos pelo Medicare nos EUA. A análise incluiu 66.974 pacientes com FEVE ≤ 35% e implante de CDI para prevenção primária, com idade média de 75 anos. Durante um acompanhamento médio de 1,4 anos, 11.876 pacientes morreram. A mortalidade em 3 anos foi de 27% em pacientes sem hospitalização por insuficiência cardíaca antes do implante de CDI, enquanto a mortalidade em 3 anos foi de 63% em pacientes com 3 ou mais hospitalizações (n = 1.263; *hazard ratio*: 1,8; intervalo de confiança de 95%: 1,6 a 2,0).^11^

Em nosso estudo, idade, BNP, FEVE e hospitalização devido à descompensação foram determinados como fatores de risco independentes, o que está de acordo com esses estudos. Alguns pacientes morrem de causas não arrítmicas logo após o implante de CDI. Os ensaios clínicos não mostram nenhum benefício do implante de CDI em pacientes de risco muito alto. Por exemplo, no estudo SCD-HeFT,^12^ a mortalidade em 2 anos foi de 30% em pacientes no grupo com o risco 20% mais alto, e o CDI foi considerado inútil neste grupo. O estudo MADIT^13^ relatou achados semelhantes. Tudo isso sugere que um CDI é inútil para prevenção primária em pacientes com alta comorbidade. A identificação de pacientes com alta comorbidade que provavelmente não se beneficiarão de um CDI é muito importante para evitar um procedimento invasivo desnecessário.

Embora a sobrevivência de pacientes isquêmicos tenha sido numericamente pior do que a de pacientes não isquêmicos, nenhuma diferença estatisticamente significativa foi encontrada. O nível de recomendação para implantação de CDI para prevenção primária em pacientes com insuficiência cardíaca não isquêmica foi rebaixado nas diretrizes, mas continua sendo recomendado com indicação de classe 1 em pacientes com insuficiência cardíaca isquêmica.^1^ Em estudos que mostram que os CDI são úteis para prevenção primária em insuficiência cardíaca isquêmica, pacientes com alto risco de arritmias foram identificados por testes eletrofisiológicos, e a taxa de terapia médica ideal recebida pelos pacientes foi baixa. No entanto, testes eletrofisiológicos pré-implantação não são realizados na prática diária. Além disso, com a disponibilidade de INRA e inibidores de SGLT2 na prática clínica, pacientes com insuficiência cardíaca isquêmica podem não mais se beneficiar da implantação de CDI para prevenção primária.

### Limitações

Apesar da experiência de diferentes operadores ao longo dos anos e do alto número de casos, o fato de que este foi um estudo unicêntrico se destaca como uma limitação.

Em nosso estudo retrospectivo, não houve equivalência estatística entre os grupos. O implante de CDI foi realizado em pacientes com um perfil pior.

## Conclusão

De acordo com os resultados do nosso estudo, o implante de CDI, além do tratamento atual para insuficiência cardíaca, não reduziu a mortalidade por todas as causas. As recomendações das diretrizes podem ser revisadas com base em estudos futuros realizados com populações de pacientes nas quais novas terapias sejam usadas juntas; portanto, devem ser conduzidos ensaios clínicos randomizados multicêntricos.
