# Supplementary material for: Desfechos Clínicos e Mortalidade em Pacientes com Cardioversor-Desfibrilador Implantável para Prevenção Primária
Source: Arq Bras Cardiol. 2025 Feb 11;122(2):e20240348. [Article in Portuguese] doi: 10.36660/abc.20240348 (PMC11870021; doi:10.36660/abc.20240348)
Supplement: Translation

# Translation: Clinical Outcomes and Mortality in Patients with Implantable Cardioverter-Defibrillator for Primary Prevention

**DOI:** 10.36660/abc.20240348i

**Authors:** Ahmet Anıl Başkurt, Sema Güneri, Reşit Yiğit Yılancıoğlu, Oğuzhan Ekrem Turan, Emin Evren Özcan

**Affiliations:** 1 Türkiye Department of Cardiology, Bakırçay University Faculty of Medicine, İzmir – Türkiye; 2 Türkiye Department of Cardiology, Dokuz Eylul University Faculty of Medicine, İzmir – Türkiye

**Keywords:** Implantable Defibrillators, Heart Failure, Primary Prevention

## Abstract

**Background:**

Implantable cardioverter-defibrillator (ICD) is indicated for primary prevention in patients with left ventricular ejection fraction (LVEF) ≤ 35% and New York Heart Association class II or III heart failure despite 3 months of optimal medical therapy. However, studies that support this recommendation are over 20 years old, and they may not reflect modern heart failure patients’ characteristics.

**Objectives:**

Retrospectively evaluate patients who received an ICD for primary prevention.

**Methods:**

All-cause and sudden death rates were compared in patients who received ICD between January 1, 2015 and March 1, 2020 and those who did not accept ICD. Variables were analyzed at a 95% confidence interval, and p < 0.05 was considered as significant.

**Results:**

When comparing mortality rates between patients with and without ICD, 67 of 228 patients (29.4%) in the ICD group and 39 of 150 patients (26%) in the control group died from all causes (p = 0.473). Age, LVEF, BNP value, and hospitalization were found to be independent predictors of all-cause mortality. Patients with BNP above 508.5, LVEF below 24.5%, and age over 68.5 years had a 25-fold increased all-cause mortality. Coronary artery disease was not found to be an independent risk factor. Survival in the control group was statistically significantly better in the first months. Although there was no statistical difference in the long term, survival was numerically better in the ICD arm. This could be attributed to the fact that ICD implantations were performed on patients with worse clinical conditions. The higher survival rate observed in patients with ICD may be due to the fact that they came in for device control and remained in follow-up.

**Conclusions:**

With advances in the treatment of heart failure, ICD implantation should be performed in selected patients.

## Introduction

Patients with left ventricular ejection fraction (LVEF) below 35% and New York Heart Association (NYHA) class II or III heart failure, after at least 3 months of optimal medical therapy, should consider primary prevention with an implantable cardioverter-defibrillator (ICD).^1^ However, the studies on which this recommendation is based are more than 20 years old, and they may not reflect the characteristics and treatment of heart failure patients today. Therefore, the effects of ICD on primary prevention may have changed.

Shock rates and mortality have decreased in patients with ICD implantation due to the development of the healthcare system, easier access to physicians, and the availability of new treatments for heart failure. With the new treatment options available for heart failure, overall mortality in this patient group has gradually decreased. Furthermore, ICD implantation for primary prevention has been questioned following heart failure trials using SGLT2 inhibitors and ARNI.^2-4^ Today, many patients with ICD do not receive any shocks. The aim of our study was to retrospectively evaluate patients who had ICD implanted for primary prevention.

## Materials and methods

We used tertiary university hospital archives, patient records, and epicrisis information from the Probel system. In this context, we screened 504 patients who underwent ICD implantation from January 2015 to March 2020 and for whom we obtained complete data. We determined that 289 of these patients received ICD implants for primary prevention. Genetic and diagnostic tests revealed that 12 of these 289 patients had channelopathy, and 49 patients underwent generator replacement. Therefore, 228 patients had heart failure with low LVEF and underwent ICD implantation for primary prevention. During the same period, the study included 150 patients as a control group who had an indication for ICD implantation for primary prevention but did not accept the treatment. The Dokuz Eylül University Non-invasive Research Ethics Committee approved the study protocol (approval number: 2020/18-02, date: August 10, 2020).

### Statistical analysis

Data were evaluated using SPSS 22.0 (IBM Corporation, Armonk, NY, USA). The normal distribution of variables was evaluated with the Kolmogorov–Smirnov test, and the homogeneity of variance was evaluated with the Levene test. Data determined by measurement were given as mean and standard deviation for those with normal distribution and as median and interquartile range for those that were not normally distributed. The unpaired t test or Mann–Whitney U test (for BNP value, hemoglobulin, creatinin) was used in the statistical analysis of these data according to the normality of the data. Categorical variables were shown as absolute and relative frequencies, and the χ^2^ test or Fisher’s exact test (for sudden death) was used, as appropriate. Variables were analyzed at a 95% confidence interval, and p < 0.05 was considered as significant. Receiver operating characteristic (ROC) analysis was used for area under the curve calculations. Multinomial logistic regression was used to determine independent predictors.

## Results

Of 228 patients with ICD for primary prevention, 175 (76.8%) were male. The mean age of the patients was 65.63 (11.94) years. The mean follow-up period was 39.45 (18.89) months. The mean duration of hospitalization for the procedure was 5.49 (3.99) days. Table 1 summarizes the patients’ demographic characteristics.

**Table 1:** – Patients’ demographic characteristics

	ICD group (n = 228)	Control group (n = 150)	p value
**Age, years, mean ± SD**	65.63 (11.94)	66.55 (12.78)	0.476
**Sex, male, n(%)**	175 (76.8%)	107 (71.3%)	0.236
**Coronary artery disease, n(%)**	135 (59.2%)	97 (64.7%)	0.286
**Hypertension, n(%)**	145 (63.6%)	106 (70.7%)	0.155
**Diabetes mellitus, n(%)**	79 (34.6%)	45 (30.0%)	0.346
**Chronic kidney disease, n(%)**	52 (22.8%)	26 (17.3%)	0.198
**COPD, n(%)**	22 (9.6%)	9 (6.0%)	0.206
**Atrial fibrillation, n(%)**	60 (26.3%)	25 (16.7%)	**0.028**
**Follow-up, months, mean ± SD**	39.45 (18.89)	38.89 (11.61)	0.724
**Beta blocker , n(%)**	222 (97.4%)	144 (96.0%)	0.552
**ACEI, ARB, ARNI, n(%)**	189 (82.9%)	130 (86.7%)	0.323
**MRA, n(%)**	182 (79.8%)	127 (84.7%)	0.233

*ACEI: angiotensin-converting enzyme inhibitor; ARB: angiotensin receptor blocker; ARNI: angiotensin receptor-neprilysin inhibitor; COPD: chronic obstructive pulmonary disease; ICD: implantable cardioverter-defibrillator; MRA: mineralocorticoid receptor antagonist; SD: standard deviation.*

Analyzing the transthoracic echocardiographic findings of patients with ICD revealed a mean LVEF of 24.30% (6.19%). On the other hand, in the control group, the mean LVEF was 30.77% (4.87%). Tables 2 and 3 display the patients’ echocardiographic findings and laboratory data.

**Table 2:** – Patients’ echocardiographic data

	ICD group (n = 228)	Control group (n = 150)	p value
**LVEF, % ± SD**	24.30% (6.19)	30.77% (4.87)	**< 0.0001**
**LVEDD, cm ± SD**	6.00 (0.81)	5.54 (0.70)	**< 0.0001**
**LVESD, cm ± SD**	5.00 (0.91)	4.17 (0.92)	**< 0.0001**
**LA, cm ± SD**	4.53 (0.62)	4.31 (0.69)	**< 0.001**
**sPAP, mmHg ± SD**	31.69 (20.09)	32.93 (15.72)	0.505

*ICD: implantable cardioverter-defibrillator; LA: left atrium; LVEDD: left ventricular end-diastolic diameter; LVEF: left ventricular ejection fraction; LVESD: left ventricular end-systolic diameter; SD: standard deviation; sPAP: systolic pulmonary artery pressure.*

**Table 3:** – Patients’ laboratory data

	ICD group (n = 228)	Control group (n = 150)	p value
**Hemoglobin gr/dL**	12.77 (1.80)	12.91 (1.93)	0.467
**Creatinine mg/dL**	1.04 (0.62)	0.89 (0.71)	**0.001**
**Na (mmol/L)**	137.74 (2.87)	138.53 (3.61)	**0.025**
**K (mmol/L)**	4.36 (0.50)	4.15 (0.46)	**< 0.0001**
**BNP pg/m**	421.00 (114.68)	415.50 (719.17)	0.932

*BNP: B-type natriuretic peptide; ICD: implantable cardioverter-defibrillator; K: potassium; Na: sodium.*

When comparing mortality rates between patients with and without ICD, 67 of 228 patients (29.4%) in the ICD group and 39 of 150 patients (26%) in the control group died from all causes (p = 0.473). We found that 2 patients in the ICD group and 8 patients in the control group had sudden death (p = 0.017; Tables 4 and 5).

**Table 4:** – All-cause mortality in ICD and control groups

		Control group (n = 150)	ICD group (n = 228)
**Mortality**	Survived	111 (74%)	161 (70%)
	Died	39 (26%)	67 (30%)
**Total**		150	228

*ICD: implantable cardioverter-defibrillator.*

**Table 5:** – Sudden death in ICD and control groups

		Grupo controle n = 150	Grupo CDI n = 228
**Sudden death**	Yes	8 (5.3%)	2 (0.9%)
	No	142 (94.7%)	226 (99.1%)
**Total**		150	228

*ICD: implantable cardioverter-defibrillator.*

We analyzed the predictors of all-cause mortality in ICD implanted patients. There was no statistically significant difference between patients with and without mortality in terms of sex or coronary artery disease. Age, diabetes mellitus and chronic renal failure were statistically significantly higher in patients with mortality (Table 6).

**Table 6:** – Demographic data of ICD patients according to survival or death

	Patients who died n = 67	Patients who survived n = 161	p value
**Age, years, mean ± SD**	70.68 (10.51)	63.57 (11.9)	**< 0.0001**
**Sex, male, n(%)**	50 (74.6%)	125 (74.6%)	0.624
**Coronary artery disease, n(%)**	45 (67.2%)	90 (55.9%)	0.115
**Hypertension, n(%)**	45 (67.2%)	100 (62.1%)	0.470
**Diabetes mellitus, n(%)**	30 (44.8%)	49 (30.4%)	**0.038**
**Chronic kidney disease, n(%)**	24 (35.8%)	28 (17.4%)	**0.003**
**COPD, n(%)**	7 (10.4%)	15 (9.3%)	0.792
**Atrial fibrillation, n(%)**	27 (40.3%)	33 (20.5%)	**0.002**
**Hospitalization for decompensated HF, n(%)**	28 (41.8%)	23 (14.3%)	**< 0.0001**
**NYHA class > 2, n(%)**	26 (38.8%)	28 (17.4%)	**0.001**

*COPD: chronic obstructive pulmonary disease; HF: heart failure; ICD: implantable cardioverter-defibrillator; NYHA: New York Heart Association.*

Upon examining transthoracic echocardiographic parameters and laboratory results, we found that patients with mortality exhibited lower LVEF, larger left atrial size, and higher systolic pulmonary artery pressure (Table 7). There was a statistically significant difference between the median BNP value between groups (Table 8).

**Table 7:** – Echocardiographic data of ICD patients according to survival or death

	Patients who died n = 67	Patients who survived n = 161	p value
**LVEF, % ± SD**	21.75 (5.47)	25.36 (6.2)	**< 0.0001**
**LVEDD, cm ± SD**	6.1 (0.70)	5.9 (0.84)	0.077
**LVESD, cm ± SD**	5.24 (0.86)	4.91 (0.91)	**0.013**
**LA, cm ± SD**	4.80 (0.58)	4.4 (0.60)	**< 0.0001**
**sPAP, mmHg ± SD**	36.75 (23.73)	29.59 (18.02)	**0.029**

*ICD: implantable cardioverter-defibrillator; LA: left atrium; LVEDD: left ventricular end-diastolic diameter; LVEF: left ventricular ejection fraction; LVESD: left ventricular end-systolic diameter; SD: standard deviation; sPAP: systolic pulmonary artery pressure.*

**Table 8:** – Laboratory data of ICD patients according to survival or death

	Patients who died n = 67	Patients who survived n = 161	p value
**Hemoglobin gr/dL**	12.29 (2.0)	13.0 (1.70)	**0.009**
**Creatinine mg/dL**	1.36 (0.61)	1.12 (0.61)	**< 0.0001**
**Na (mmol/L)**	137.00 (2.80)	138.06 (2.85)	**0.008**
**K (mmol/L)**	4.38 (0.57)	4.36 (0.48)	0.877
**BNP pg/m (n = 177)**	1424.44 (1384.83)	537.82 (783.80)	**< 0.0001**

*BNP: B-type natriuretic peptide; ICD: implantable cardioverter-defibrillator; K: potassium; Na: sodium.*

### Predictors of mortality in multinomial logistic regression analysis

Multinomial logistic regression analysis included variables that may influence mortality in the model using the “enter” method. These variables included age, coronary artery disease, diabetes mellitus, chronic renal failure, basal rhythm, hospitalization for heart failure, NYHA class > 2, complications, and BNP value. The analysis revealed that age, LVEF, BNP value, and hospitalization for decompensation were independent factors for all-cause mortality. Mortality was 3.4 times higher in patients hospitalized with decompensated heart failure before the procedure. Coronary artery disease was not an independent risk factor (Table 9).

**Table 9:** – Multinomial logistic regression analysis results

	B	SE	Wald	OR (95% CI)	p
**Age**	0.067	0.021	9.799	1.069 (1.025 a 1.114)	**0.002**
**LVEF**	–0.097	0.036	7.411	0.907 (0.846 a 0.973)	**0.006**
**CAD**	0.032	0.409	0.006	1.033 (0.463 a 2.301)	0.937
**DM**	0.530	0.397	1.782	1.699 (0.780 a 3.698)	0.182
**CKD**	–0.181	0.484	0.140	0.834 (0.323 a 2.155)	0.709
**AF**	0.306	0.414	0.547	1.358 (0.603 a 3.060)	0.460
**Hospitalization for decompensated HF**	1.211	0.433	7.820	3.355 (1.436 a 7.839)	**0.005**
**NYHA class > 2**	0.007	0.447	0.000	1.007 (0.420 a 2.418)	0.987
**BNP**	0.001	0.000	6.175	1.001 (1.000 a 1.001)	**0.013**

*AF: atrial fibrillation; BNP: B-type natriuretic peptide; CAD: coronary artery disease; CI: confidence interval; CKD: chronic kidney disease; DM: diabetes mellitus; LVEF: left ventricular ejection fraction; NYHA: New York Heart Association; OR: odds ratio; SE: standard error.*

We performed ROC analysis for these variables, finding predictive values of 68.5 years with 62% sensitivity and 62% specificity for age, 24.5% with 54% sensitivity and 63% specificity for LVEF, and 508.5 with 69% sensitivity and 69% specificity for BNP value.

The all-cause mortality of patients with BNP value above 508.5, LVEF value below 24.5%, and age greater than 68.5 years was 25 times higher than the other patients (Table 10).

**Table 10:** – Univariate logistic regression analysis results

	B	SE	Wald	OR (95% CI)	p
**Age < 68.5 years BNP < 508.5 LVEF > 24.5%**	–3.243	1.029	9.938	0.039 (0.005 a 0.293)	0.002

*BNP: B-type natriuretic peptide; CI: confidence interval; LVEF: left ventricular ejection fraction; OR: odds ratio; SE: standard error.*

When the survival curves of the two groups were evaluated, survival in the control group was statistically significantly better in the first months compared to the ICD implanted group. At month 44, the survival curves of the two groups were crossed. Although there was no statistical difference in the long term, survival was numerically better in the ICD arm. Mean survival was 59 months in the ICD implanted group and 55 months in the control group (Figure 1).

**Figure 1:** – Survival in ICD and control groups. ICD: implantable cardioverter-defibrillator.

This could be attributed to the fact that ICD implantations were performed on patients with worse clinical conditions. In the long term, we believe that ICD implanted patients had a higher survival rate because they came in for device control and remained in follow-up.

## Discussion

While there was no significant difference between the ICD implanted patients and the control group in terms of all-cause mortality, a statistically significant difference was observed in terms of sudden death (p = 0.017). Sudden death was observed in 2 patients in the ICD arm and in 8 patients in the control group, and this statistical difference may be due to the low number of events.

A published meta-analysis examined 12 randomized trials of heart failure with low LVEF over a 20-year period from 1995 to 2014 for sudden cardiac death risk. A total of 40,195 patients were included in the studies. The annual incidence of sudden cardiac death was 6.5% in RALES,^5^ the first study covering this period, and 3.3% in the most recent PARADIGM-HF study.^6^ There was a 44% reduction in the rate of sudden cardiac death over a 20-year period (hazard ratio 0.56; 95% confidence interval 0.33 to 0.93; p = 0.03). The 90-day cumulative incidence of sudden cardiac death was 2.4% in older studies and 1.0% in recent studies.^7^

When we analyzed the studies performed in a similar manner to our study, we found that 45,000 patients with ICD for primary prevention were studied in the USA. The study identified the following 7 mortality predictors, which were abbreviated with the letters “SHOCKED”: age ≥ 75 years, heart failure, atrial fibrillation, chronic obstructive pulmonary disease, chronic kidney disease, LVEF ≤ 20%, and diabetes. In the model test group, using the SHOCKED model, 3-year mortality was 65% in patients in the top 10% risk group.^8^ Another study published in 2012 included 2717 patients. In this publication, peripheral arterial disease, age ≥ 70 years, creatinine > 2 mg/dL and LVEF ≤ 20% were determined as risk criteria. In patients with ≥ 3 of these risk criteria, mortality was found to be 4 times higher (16.5% versus 3.4%) compared to patients with < 3 criteria.^9^ A similar study was published in 2012. Using 900 patients, a score called FADES was developed. In this score, NYHA > III, advanced age, diabetes mellitus, LVEF ≤ 25%, and smoking were found as risk criteria.^10^The results of our study are similar to these studies.

Another study examined the impact of heart failure burden and comorbid condition burden on survival in Medicare patients with ICD for primary prevention in the USA. The analysis included 66,974 patients with LVEF ≤ 35% and ICD implantation for primary prevention, with a mean age of 75 years. During a mean follow-up of 1.4 years, 11,876 patients died. Three-year mortality was 27% in patients with no hospitalization for heart failure before ICD implantation, whereas 3-year mortality was 63% in patients with 3 or more hospitalizations (n = 1,263; hazard ratio 1.8; 95% confidence interval 1.6 to 2.0).^11^

In our study, age, BNP, LVEF, and hospitalization due to decompensation were determined as independent risk factors, which is in agreement with these studies. Some patients die of non-arrhythmic causes shortly after ICD implantation. Clinical trials show no benefit of ICD implantation in very high-risk patients. For example, in the SCD-HeFT^12^ study, 2-year mortality was found to be 30% in patients in the top 20% risk group and ICD was found to be useless in this group. The MADIT^13^ study reported similar findings. All this suggests that an ICD is useless for primary prevention in patients with high comorbidity. Identification of patients with high comorbidity who are unlikely to benefit from an ICD is very important to avoid an unnecessary invasive procedure.

Although the survival of ischemic patients was numerically worse than non-ischemic patients, no statistically significant difference was found. The recommendation level for ICD implantation for primary prevention in patients with non-ischemic heart failure has been downgraded in the guidelines, but it continues to be recommended with class 1 indication in patients with ischemic heart failure.^1^ In studies showing that ICD are useful for primary prevention in ischemic heart failure, patients at high risk of arrhythmias were identified by electrophysiological testing, and the rate of optimal medical therapy received by patients was low. However, pre-implantation electrophysiological tests are not performed in daily practice. Furthermore, with the availability of ARNI and SGLT2 inhibitors in clinical practice, patients with ischemic heart failure may no longer benefit from ICD implantation for primary prevention.

### Limitations

Despite the experience of different operators over the years and the high number of cases, the fact that this was a single-center study stands out as a limitation.

In our retrospective study, there was no statistical equivalence between the groups. ICD implantation was performed in patients with a worse profile.

## Conclusion

According to the results of our study, ICD implantation in addition to current heart failure treatment did not reduce all-cause mortality. Guideline recommendations can be revised with studies performed with patient populations in which new therapies are used together in the future; therefore, multicenter randomized controlled trials should be conducted.
